# An Evaluation of the Effect of the Use of N95 Respirators by Surgical Teams on Early Surgical Site Infections in Orthopedic Cases

**DOI:** 10.7759/cureus.25138

**Published:** 2022-05-19

**Authors:** Ercan Hassa, Taner Alıç

**Affiliations:** 1 Orthopaedics and Traumatology, Hitit University Corum Erol Olcok Training and Research Hospital, Corum, TUR

**Keywords:** orthopedics-related infections, covid-19 outbreak, medical mask, n95 respirator, surgical site infections

## Abstract

Background

Surgical site infections (SSIs) are seen in the postoperative period in orthopedic and traumatology clinics. Just as in all surgical clinics, SSIs lead to patient dissatisfaction with the results, prolong the length of stay in the hospital, and increase treatment costs. SSIs are known to occur as a result of wound contamination through inoculation of microorganisms found mainly in the air or in the surgical area. Because of the coronavirus disease 2019 pandemic, N95 masks have been widely used in the operating rooms of our hospital by nurses, residents, and surgeons since March 2020. This study aims to evaluate the effect of N95 respirator use by the surgical team on SSIs determined in patients operated on in our clinic compared to surgical mask use.

Methodology

In this retrospective study, the use of N95 respirators by the surgical team was compared with the use of surgical masks to evaluate the effect on SSIs in patients operated on in our clinic. Two groups were formed of patients operated on by the surgical team wearing surgical masks between February 2019 and February 2020 and those operated on with the surgical team using N95 respirators between March 2020 and March 2021. Each patient was diagnosed with postoperative SSIs by two different surgeons in the same clinic and by an infection clinic specialist based on clinical and laboratory findings.

Results

A total of 1,486 patients were examined; 729 patients in February 2019-February 2020 period (Group 1) and 757 in March 2020-March 2021 period (Group 2). In total, 124 and 104 patients were excluded from the first and second groups, respectively, for various reasons, including revision surgery, open fractures, diabetes, smoking, peripheral vascular disease, or other comorbidities that could affect infection rates. SSIs were determined in 35 patients in Group 1 and 13 patients in Group 2. The SSI rates in the second period in both types of procedures (arthroplasty and trauma surgeries) were determined to be significantly lower.

Conclusions

Because of the use of intraoperative N95 respiratory masks by surgical teams in orthopedics and traumatology procedures, the number of SSIs decreased significantly compared to the use of surgical masks.

## Introduction

Surgical site infections (SSIs) constitute a significant proportion of hospital-acquired infections in surgical patients [1]. Patients with SSIs comprise 14-16% of all hospitalized patients with nosocomial infections [2]. SSIs are defined as infections originating within 30 days postoperatively or microbial contamination of the surgical wound within the first year postoperatively if an implant has been applied to the patient [3]. SSIs in orthopedic practice present a challenging situation for both patients and surgeons [4]. Because SSIs require long-term antibiotics, have high morbidity and mortality rates, and prolong the length of stay in the hospital, they pose an additional economic burden on hospital resources [5].

SSIs related to orthopedic procedures can extend patients’ length of stay in the hospital by up to two weeks. The need for repeated debridement increases costs and significantly reduces rehabilitation capacity. As the outcomes can cause physical limitations, this is a severe and catastrophic complication for both patients and surgeons. The incidence of orthopedic SSIs can range between 0.8% and 71% [6]. Comorbidities known to increase the risk of SSIs include smoking, malnutrition, diabetes, immune disorders, malignancy, and prolonged operating time [7].

Following total knee arthroplasty (TKA), deep infection is one of the worst complications with respect to the high cost of inpatient treatment [8]. Infection after TKA has been reported at a frequency of 0.5-2%, which increases in patients with chronic steroid use, immune disorders, or diabetes [9]. In a study of 16,035 primary TKA cases, Hanssen and Rand [10] reported that infection developed with an incidence of 2%, and in 2,714 patients with revision surgery, at a rate of 5.6%. Patient-related factors, operating room conditions (laminar airflow, correct cleaning, and draping of the appropriate area), surgical technique, the materials used, and postoperative follow-up play an important role in developing infections following TKA.

Factors that can cause deep infection in the postoperative period include rheumatoid arthritis [11], diabetes mellitus (HbA1c >7%) [12,13], a history of malignancy [13], the presence of human immunodeficiency virus (CD4 30) [14], smoking [12,13], intravenous substance addiction [15], organ transplantation-related corticosteroid use [16], a history of knee surgery [17], a history of septic arthritis or osteomyelitis [18], prolonged operating time [12], malnutrition [19], skin problems [13], and a history of intra-articular steroid injections [20].

In December 2019, a new coronavirus was reported in Wuhan, China, causing coronavirus disease 2019 (COVID-19). The virus rapidly spread across many countries and was declared a global pandemic in March 2020. To date, more than 150 million people worldwide have been infected and there have been more than 3.5 million deaths. As protection against the virus, the measures of social distancing and wearing face masks were implemented globally. Moreover, in healthcare institutions, those in close contact with the disease started using N95 respirators. With the onset of the pandemic, the protective measures in the healthcare system in Turkey included the use of N95 respirators by healthcare personnel, especially those in intensive care units, infection clinics, and in wards treating COVID-19 patients.

Surgical or medical masks are single-use masks that sit loosely on the face forming a physical barrier between potential pollutants in the environment and the nose and mouth of the wearer. Surgical masks are produced with varying thicknesses and properties to protect against contact with fluids. When worn correctly, a surgical mask helps to prevent the spread to the airway of large droplets which may contain viruses or bacteria. The use of surgical masks also aims to reduce the spread of saliva and respiratory expression to the airway. However, the design cannot filter or prevent some very small particles in the air. In addition, surgical masks cannot provide full protection because of the loose fit between the mask surface and the face.

The N95 respirator has been designed to fit very closely to the face not allowing any leakage and to provide very efficient filtration of fine particles in the air. All N95 respirators approved by the Food and Drug Administration are labeled as single-use devices. The clinical effectiveness of N95 respirators and surgical (medical) masks in the prevention of respiratory tract infections has not been fully evaluated. Quantitative protection analysis of healthcare personnel in close contact with patients with suspected respiratory tract disease has remained limited to a great extent [21]. A study reported that the use of surgical masks in clean surgical practices did not affect SSIs [22].

This retrospective study aimed to evaluate the effects of the use of N95 respirators on SSIs in patients who had undergone orthopedic and traumatology surgery. Surgical teams started to use the masks in operating room conditions because of the COVID-19 pandemic.

## Materials and methods

**Figure 1:**
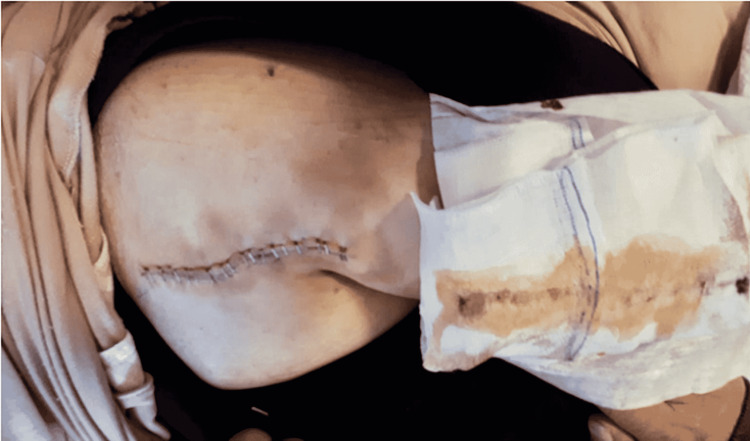
Serous discharge from an infected patient who underwent surgery in our clinic.

**Figure 2:**
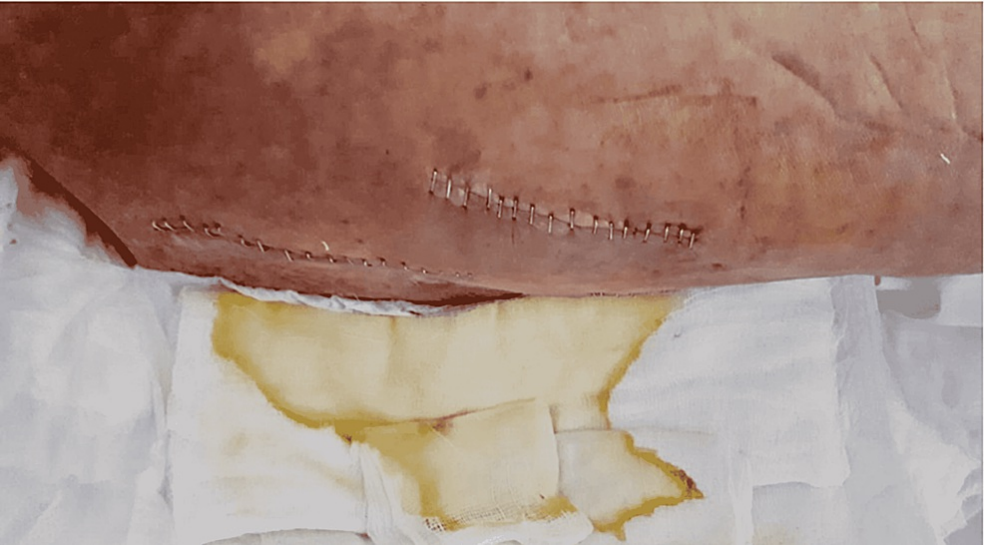
Seropurulent discharge from an infected patient who underwent surgery in our clinic.

**Figure 3:**
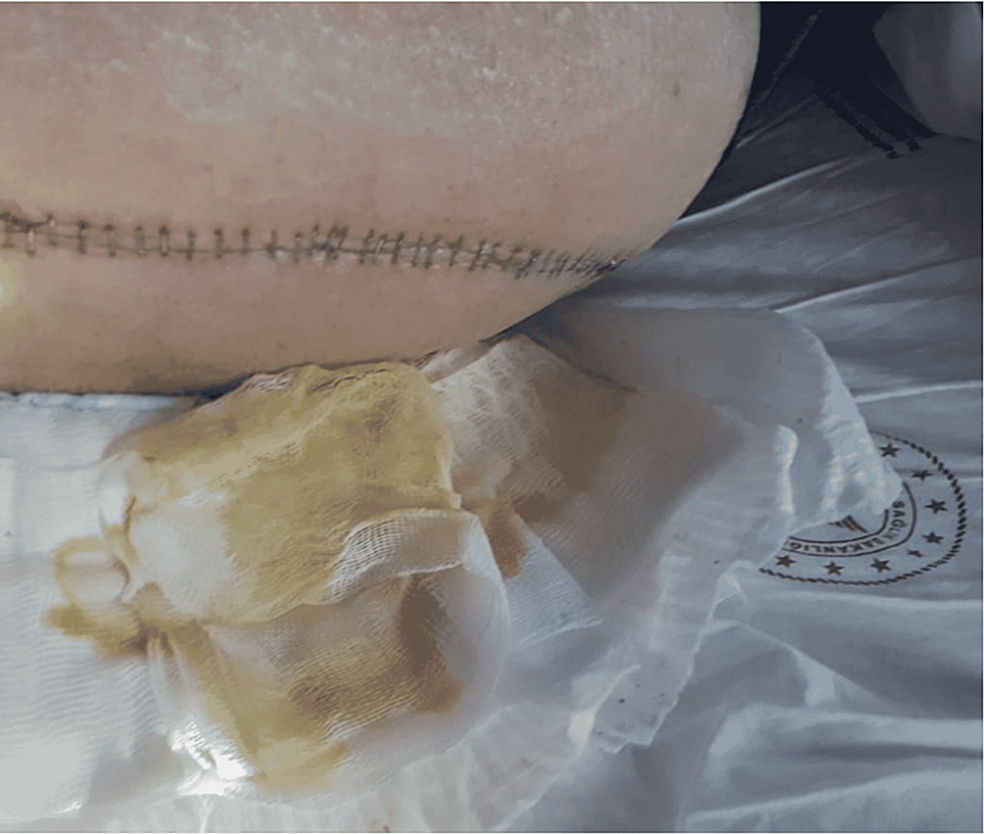
Purulent discharge from an infected patient who underwent surgery in our clinic.

This study aimed to evaluate the two different mask types (medical mask and N95 respirator) used by surgical teams and the rate of early postoperative wound infection in patients who underwent surgery in the Orthopaedics and Traumatology Clinic of our hospital between February 2019 and March 2021. The diagnosis of SSIs was made at the end of the second week using the findings of C-reactive protein (CRP) >5 mg/L and the presence of redness and/or serous (Figure 1), seropurulent (Figure 2), or purulent (Figure 3) discharge in the wound. Exclusion criteria included malnutrition, peripheral vascular disease, oncological disease, diabetes, a history of immunosuppressive therapy, rheumatological disease, or a history of infection in a surgical site.

From a total of 729 patients operated on from February 2019 to February 2020 with the surgical team wearing medical face masks, 124 patients were excluded. The remaining 605 patients (305 males and 300 females) comprised Group 1. From a total of 757 patients operated on from March 2020 to March 2021 with the surgical team using N95 respirators, 104 patients were excluded. The remaining 653 patients (334 males and 319 females) comprised Group 2.

The operating rooms for orthopedics and traumatology patients in our hospital were selected as they are equipped with HEPA filters. Standard sterile area cleaning and draping were performed for all surgical patients. Tourniquet was not applied to any of the patients examined in this study. Prophylactic antibiotic, intravenous (IV) cefazolin (first-generation cefalosporin), at a dosage adjusted per kilo was administered 45 minutes before the operation. All operations studied (arthroplasty patients, trauma patients without open skin wounds, and elective surgery patients) were recorded with a fixed number of staff consisting of one surgeon and three surgical nurses.

Statistical analyses

Data obtained in the study were analyzed statistically using SPSS software version 22.0 (IBM Corp., Armonk, NY, USA). Conformity of the data to normal distribution was assessed using the Shapiro-Wilk test. Descriptive statistics were recorded as mean ± standard deviation (SD) or median (minimum-maximum) values according to the data distribution. Categorical data were presented as number (n) and percentage (%). To compare numerical data between two independent groups, the Student’s t-test was used for data with normal distribution, otherwise, the Mann-Whitney U-test was applied. The chi-square test was used to compare categorical variables. A p-value of <0.05 was considered statistically significant.

## Results

Group 1 included 605 patients comprising 305 males and 300 females with a mean age of 64.4 ± 19.92 years. All patients in Group 1 were operated on between February 2019 and February 2020 by surgical teams using medical masks. All procedures (arthroplasty and trauma patients without open skin wound components and elective surgeries) in this group were shorter than one hour. In 35 patients who were evaluated as infected, the mean postoperative length of hospital stay was 16.71 ± 10.46 days, and the mean CRP value was 53.48 ± 26.36 mg/L. The operations in these 35 patients were arthroplasty in 14, and trauma and other elective surgeries in 21. Purulent discharge was noted in three patients, seropurulent discharge in eight, and serous discharge in 24. Redness in the operated region was observed in all patients. SSI was diagnosed at a rate of 5.4% in the arthroplasty subgroup of Group 1 and at 6.0% in the trauma and elective surgery subgroup. In Group 1, debridement of the surgical area because of deep infection was performed among 21% of arthroplasty patients with SSI and 10% of non-arthroplasty patients with SSI.

**Figure 4:**
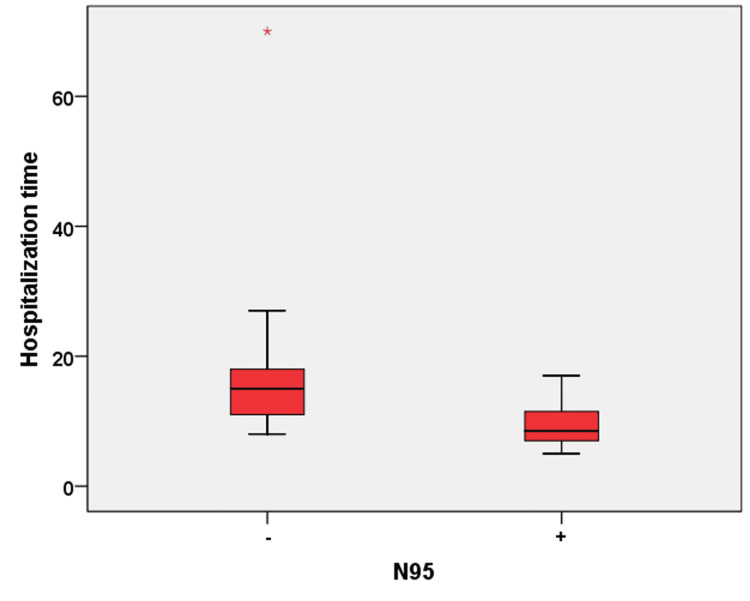
Graphic representation of the CRP values and length of stay in the hospital among patients with SSIs according to the use of N95 respirators. CRP: C-reactive protein; SSI: surgical site infection

Group 2 included 653 patients comprising 334 males and 319 females with a mean age of 68.69 ± 20.87 years. All patients in Group 2 were operated on between March 2020 and February 2021 by surgical teams using N95 respirators. All procedures (arthroplasty and trauma patients without open skin wound components and elective surgeries) in this group were shorter than one hour. No statistically significant difference was noted between the subgroups regarding age, operating time, and preoperative anesthesia evaluation scores (American Society of Anaesthesiologists, ASA), which could affect infection rates (p = 0.174). The diagnosis of SSIs was made at the end of the second week using CRP levels of >5 mg/L, as well as the presence of redness and/or serous, seropurulent, or purulent discharge in the wound. In 13 patients evaluated as infected, the mean postoperative length of hospital stay was 8.46 ± 2.33 days, and the mean CRP value was 30.15 ± 12.78 mg/L. The postoperative length of stay and CRP values at the end of two weeks were determined to be statistically significantly lower in Group 2 patients who were diagnosed with SSIs compared to those in Group 1 (p < 0.001, p = 0.004, respectively). The operations in these 13 patients included arthroplasty in 2 cases, and trauma and other elective surgeries in 11. Purulent discharge was determined in one patient, seropurulent discharge in one, and serous discharge in 10 patients. Redness in the operated region was observed in all patients. SSI was diagnosed at a rate of 1.3% in the arthroplasty subgroup of Group 2 and at 2.2% in the trauma and elective surgery subgroup. In Group 2, debridement of the surgical area because of deep infection was performed among 10% of non-arthroplasty patients with SSIs and in none of the arthroplasty subgroup patients. These SSI rates in two different surgery types were determined to be statistically significantly lower than in Group 1 (p = 0.040). Figure 4 presents the CRP values and length of hospital stay in patients with SSI and the use of N95 respirators.

In the wound site and tissue samples taken from patients with SSIs, culture growth was determined in one patient in Group 1 (*Staphyloccocus hemolyticus*) and in one patient in Group 2 (*Enterococcus faecium*). 

## Discussion

While many factors affecting the number of viable bacteria in the air of operating rooms are known [23], the difference between the effect of surgical masks and N95 respirators has not been investigated to date. Although the use of these masks and respirators in respiratory diseases has been investigated and presented in the literature [23], their effects on the surgical field have not been clarified yet. This study aimed to determine the relationship between the prevention and/or reduction of early SSIs and the use of N95 respirators instead of surgical masks by orthopedic surgical teams during the COVID-19 pandemic as a result of reducing microbial spread to the surgical area via the inoculation route. N95 respirators started to be used in healthcare institutions in Turkey in March 2020, at the same time as throughout the world, because of the COVID-19 pandemic. A retrospective investigation was done using two large series of patients operated on in our hospital in different periods with and without the use of N95 respirators.

The study findings show that the early period SSI rates in all patient groups were statistically significantly reduced with the use of N95 respirators compared to surgical masks. In addition, we observed that the use of N95 respirators in patients with early SSIs significantly reduced the infection laboratory parameters and shortened the hospital stay compared to the use of surgical masks.

In this study, several factors such as smoking, vascular disease, diabetes, malignancies, duration of surgery, use of tourniquet, and the presence of open wounds, which may be risk factors for early-stage orthopedic SSIs, were taken into account and excluded from the study. Operations were performed in the same operating room by the same surgical teams accompanied by the same prophylactic antibiotic administration. The only difference between the two groups was the type of mask used by the surgical team. The number of patients included in the study is high. The fact that the study was single-centered can be evaluated negatively. However, there is sufficient similarity between the two groups, and the diagnosis of SSIs by the same doctors can be counted as a positive factor in this study.

Deep infections requiring debridement were noted in three arthroplasty patients and in two patients who underwent non-arthroplasty surgery performed by a surgical team wearing surgical masks, and in only one patient, not an arthroplasty case, in the group where the surgical teams used N95 respirators.

In cultures taken from the wound site, there was no bacterial growth in most patients due to possible antibiotic suppression. Hence, there is a need for further detailed multicentric studies to investigate microbial growth in cultures. This can be considered a negative feature of the study.

Although no significant difference can be reported with the use of N95 respirators because of the low numbers of deep infections in the groups examined in this study (p = 0.649), significant results could be obtained with further studies among larger populations. The low production in the microbial cultures of the tissue samples taken from the patients could be due to the difficulty of determining culture growth caused by antibiotic suppression. As reported in the literature [24], decreases in SSI rates can be predicted to play a key role in reducing inpatient treatment costs. Additional studies are required to evaluate the cost of patient treatment because of SSIs and the use of N95 respirators by surgical teams.

## Conclusions

The results of this study showed that the use of N95 respirators by surgical teams reduced early postoperative SSIs after orthopedic procedures compared to surgical masks. It is reasonable to think that this effect is due to the reduced human-induced microbial load seen in the airways. The use of N95 respirators in orthopedic procedures may play an effective role in reducing hospital stay due to infections because they reduce SSI rates. We think that the use of N95 respirators, which are extremely easy to obtain, by surgical teams should be considered for these reasons.
